# Transcriptomic Profiling of Developmental Stages and Screening of Candidate Genes in *Pholiota nameko*

**DOI:** 10.3390/jof12070542

**Published:** 2026-07-22

**Authors:** Yao Zhu, Tingting Ma, Yichu Wang, Jiayi Liu, Xiaolong He, Junshen Wang, Pengfei Jin, Xiaopeng Gao

**Affiliations:** Shaanxi Key Laboratory of Research and Utilization of Resource Plants on the Loess Plateau, Engineering Research Center of Microbial Resources Development and Green Recycling of Shaanxi Province, College of Life Sciences, Yan’an Medical College Yan’an University, Yan’an 716000, China; zynt2160@163.com (Y.Z.); matt9303@163.com (T.M.); 17629194925@163.com (Y.W.); ljy55635@163.com (J.L.); ydsky_mush@163.com (X.H.); 15891783352@163.com (J.W.)

**Keywords:** *Pholiota nameko*, developmental stages, transcriptome, real-time quantitative PCR (RT-qPCR)

## Abstract

The developmental cycle of *Pholiota nameko* can be divided into four stages: the mycelial stage (JS), the primordium stage (FH), the growth stage (SZ), and the maturity stage (CS). In this study, transcriptome sequencing was performed on *P. nameko* at these four stages, followed by the screening of differentially expressed genes and functional annotation via Gene Ontology (GO) and Kyoto Encyclopedia of Genes and Genomes (KEGG) analyses. By integrating the annotation results of all differentially expressed genes (DEGs), we screened for candidate genes potentially correlated with the growth and development of *P. nameko*. The expression levels of these candidate genes were then compared using real-time quantitative PCR (RT-qPCR) to identify those with the highest expression. The results showed that in the FH vs. CS and SZ vs. CS comparisons, DEGs were mainly enriched in pathways related to protein processing, fatty acid metabolism, and linoleic acid metabolism, suggesting that alterations in these specific metabolic pathways may be closely associated with the growth and development of *P. nameko*. Through analysis based on upregulation and log_2_ fold change (log_2_FC) values, further screening identified 13 candidate genes, and the gene *Cluster-7415.13* with the highest expression level was preliminarily screened out through RT-qPCR analysis. This gene may be potentially involved in processes such as rapid cell expansion, cell wall synthesis, nutrient absorption, and active growth-stage metabolism.

## 1. Introduction

Macrofungi that produce conspicuous fruiting bodies and are deemed safe for human consumption are known as edible fungi, or edible mushrooms [1,2,3]. Rich in dietary fiber, high-quality protein [4,5], and low in fat, they serve as a daily dietary staple, as a result, they are valued by consumers for their distinctive nutritional profile [6,7]. Moreover, they are abundant in bioactive compounds, such as polysaccharides [8,9] and polyphenols [10]. Beyond basic nutrition, they exhibit significant bioactive properties and potential medicinal value, including antioxidant [11,12], antitumor [13], immunomodulatory [14], hypoglycemic [15], and antihypertensive effects [16,17].

*Pholiota nameko*, often referred to as the slippery mushroom, pearl mushroom, or smooth-capped *Pholiota*, is a member of the genus *Pholiota* and family Pholiotaceae [18,19]. Its name comes from the slimy surface of its cap [20,21,22]. The mycelial stage (JS), primordium stage (FH), growth stage (SZ), and maturity stage (CS) are the four stages of *P*. *nameko* growth [23]. During the mycelial stage, as the mycelium continuously grows in the culture medium and degrades the substrate to acquire nutrients, reserve materials are accumulated to support subsequent fruiting-body development [24]. Fruiting-body differentiation is initiated when hyphae aggregate and intertwine to form small, white, raised primordia [25]. The morphology of the fruiting bodies rapidly develops, with the cap and stem gradually taking shape, and the fruiting bodies quickly expand and elongate [20]. During the maturity stage, spores progressively mature, and the cap becomes fully expanded [26]. Transcriptome investigations of developmental stages in other edible fungi have advanced considerably. For example, the transition from mycelium to primordia in the rough-skinned wood ear involves a more complex gene regulatory network, and genes associated with cell proliferation, differentiation, and protein synthesis are predominantly enriched during primordium growth [27]. Differentiation of *Lentinula edodes* primordia is induced by aeration cues, accompanied by the activation of genes associated with cell adhesion and cell wall remodeling [28]. Significant transcriptome remodeling occurs between developmental phases, as evidenced by the fact that 19–34% of differentially expressed genes are unique to each developmental stage of *Flammulina velutipes* [29]. The most pronounced transcriptional changes are observed during the transition from spores to mycelium in *Oudemansiella radicata*, and primordium formation is associated with glycerophospholipid metabolism and GTPase signaling pathways [1]. Despite these available resources, the regulatory mechanisms underlying the growth and development of *P. nameko* remain poorly understood. In contrast, the transcriptome features of *P. nameko* at different developmental stages remain poorly characterized, and the regulatory mechanisms governing these stages are still largely unclear. Transcriptome analysis, which operates at the RNA level and aims to investigate specific gene expression profiles, is a critical technique for elucidating cellular phenotypes and biological functions [30,31,32].

In this study, transcriptome sequencing was employed to examine spatiotemporal transcriptomic changes within the fruiting bodies of *P. nameko* using samples collected from its four growth stages [33]. We examined the dynamic expression patterns of genes throughout the entire growth process of *P. nameko*, from mycelial growth to fruiting-body maturation, by identifying genes showing expression changes that correlate with phenotypic transitions, as well as the metabolic pathways that were enriched among these differentially expressed genes. Candidate genes whose expression dynamics were consistent with the developmental stages were selected and validated by real-time quantitative PCR (RT-qPCR), aiming to provide preliminary molecular-level clues regarding the potential mechanisms regulating the growth and development of *P. nameko*.

## 2. Materials and Methods

### 2.1. Test Substances

The test strain of *P. nameko* (H-9, Lemon-scaled *Pholiota*) was provided by the Edible Fungus Molecular Breeding Laboratory at the School of Life Sciences, Yan’an University. Cap samples were collected from *P. nameko* strains at the JS, FH, SZ, and CS stages. The caps were cleaned with phosphate-buffered saline (PBS) in a laminar flow hood to remove contaminants, then cut into 0.5 cm pieces and blotted dry. Three samples were taken for each stage, representing three biological replicates. The samples were placed in 4 mL centrifuge tubes, flash-frozen in liquid nitrogen for 15 min, and stored at −80 °C. Subsequently, they were shipped on dry ice to a commercial sequencing company for transcriptome analysis.

### 2.2. Experimental Methods

#### 2.2.1. Building cDNA Libraries and Workflow for Transcriptome Sequencing

Total RNA was extracted by the sequencing company, and its concentration and integrity were assessed using a Qubit 4.0 (Thermo Fisher Scientific, Waltham, MA, USA) and Qsep 400 (BiOptic Inc., New Taipei City, Taiwan, China), respectively. The Illumina platform (Illumina, Inc., San Diego, CA, USA) was used to create and sequence cDNA libraries after the samples met quality requirements.

#### 2.2.2. Analysis of Transcriptome Sequencing Information

The Fastp v0.23.2 program was used to quality-control the raw sequencing data to remove low-quality data and provide clean reads. The clean reads were then assembled using Trinity v2.8.5 to produce reference sequences for further investigation [34]. Corset v1.09 was used to cluster transcripts hierarchically according to read counts and expression patterns. The sequences were aligned against the KEGG, NR, Swiss-Prot, GO, and TrEMBL databases using the DIAMOND BLASTX software v2.1.15 to gather annotation data for additional study [35]. By integrating the annotation results of all differentially expressed genes, we screened for common differentially expressed genes across different comparison groups using the criteria of log_2_FC ≥ 1 and FDR < 0.05.

#### 2.2.3. RT-qPCR Analysis

Sangon Biotech (Shanghai, China) Co., Ltd. designed RT-qPCR primers using Premier v5.0 software based on the gene sequences obtained by sequencing. PCR was performed to validate the primers [36,37]. Total RNA was extracted from four developmental stage samples of *P. nameko* using the Plant RNA Dual-Column Kit (Polysaccharide and Polyphenol R4150) (Magen, Guangzhou, China). RNA concentration was determined with a micro-spectrophotometer (sample volume: 1 µL); first-strand cDNA was synthesized using the All-in-One qRT Mix with dsDNase (TOROBlue^®^). For subsequent use, the primers were centrifuged, diluted, mixed, aliquoted, and stored at −20 °C [38]. The RT-qPCR reaction mixture was prepared on ice. The samples were added to the 96-well plate in the order indicated by the experimental design once the RT-qPCR reaction mixture was prepared [37]; *EF1α-1* and *EF1α-2* were selected as candidate reference genes; based on Ct values from four developmental stages, TBtools v1.098 was used to integrate geNorm v3.5, NormFinder, and BestKeeper v1.0 for evaluating gene expression stability, and combined with geNorm pairwise variation and one-way ANOVA, the optimal reference gene was screened and *EF1α-1* was finally determined as the single calibrator reference gene for RT-qPCR in this study [38,39,40,41], the target gene’s relative expression levels were measured using the 2^−ΔΔCt^ technique [38,42,43], and statistical analysis was carried out using OriginPro v2021.

## 3. Results

### 3.1. Collection of P. nameko Samples

**Figure 1:**
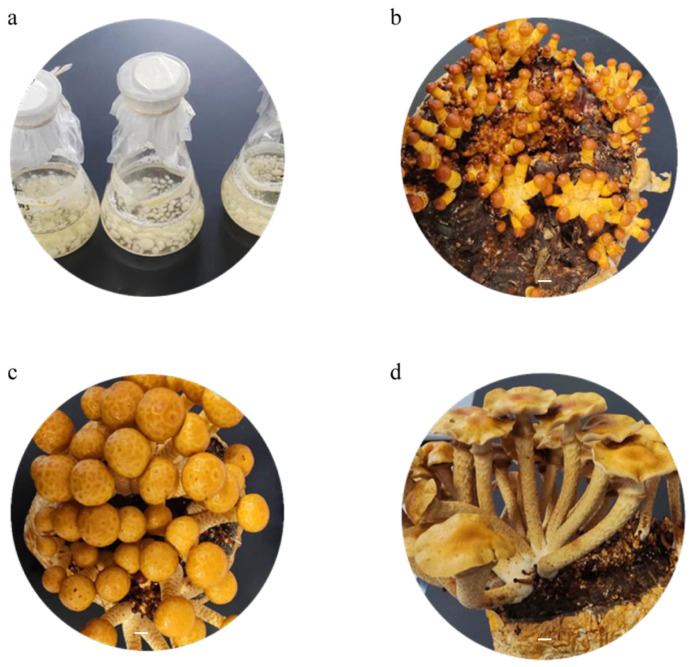
Four growth stages of the mushroom samples. (**a**) JS, (**b**) FH, (**c**) SZ, and (**d**) CS. Bar, 1 cm.

Samples of *P. nameko* at various stages: (a) mycelial stage (JS), with the mycelium first appearing white and then turning pale yellow; (b) primordium stage (FH), when structural features like the pileus and stipe start to differentiate; (c) growth stage (SZ), marked by a plump pileus and vigorous metabolic activity; (d) maturity stage (CS), when the pileus expands, spores are expelled, and growth ceases (Figure 1).

### 3.2. Comprehensive Analysis of Differentially Expressed Genes

**Figure 2:**
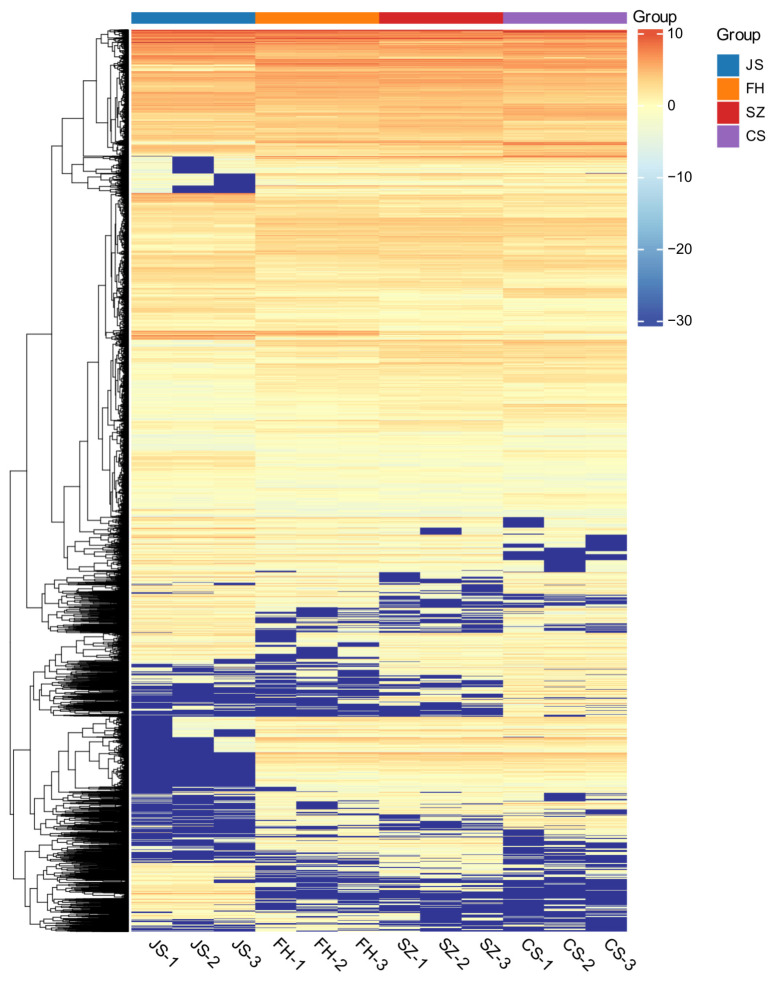
Heatmap of DEGs from four developmental stages (JS, FH, SZ, CS). The right color scale shows log_2_-transformed relative expression levels (red: high, blue: low). Rows are clustered genes with a left dendrogram; columns are biological replicates, and the top bar denotes sample groups.

The K-means time-series clustering results of normalized gene expression levels across the four developmental stages (JS, FH, SZ, and CS) are displayed (Figure 2), classifying all genes into ten distinct expression subclasses. In each panel, the solid black line represents the average expression pattern of genes within the subclass, the colored shaded area indicates the range of expression variation within the cluster, and the number of genes in each subclass is indicated above the panel. Subclasses 4 and 7 contained the largest numbers of genes, whereas subclass 3 contained the fewest. Notably, substantial variations in temporal expression patterns were observed among the different subclasses. Most genes exhibited a single expression peak, occurring at the FH and SZ stages, respectively; however, some genes displayed a bimodal pattern, with expression peaks appearing successively at the FH and CS stages. In addition, certain genes remained at low expression levels during the early stages but were significantly upregulated during the SZ and CS stages, whereas others were highly expressed at the JS stage and gradually downregulated as development progressed. Each developmental stage exhibited distinct clusters of highly expressed genes. Furthermore, the varying widths of the shaded areas reflect notable differences in the synchrony of expression dynamics among the different gene clusters. Collectively, these results demonstrate that as development progresses, genes exhibit coordinated, stage-specific expression patterns.

**Figure 3:**
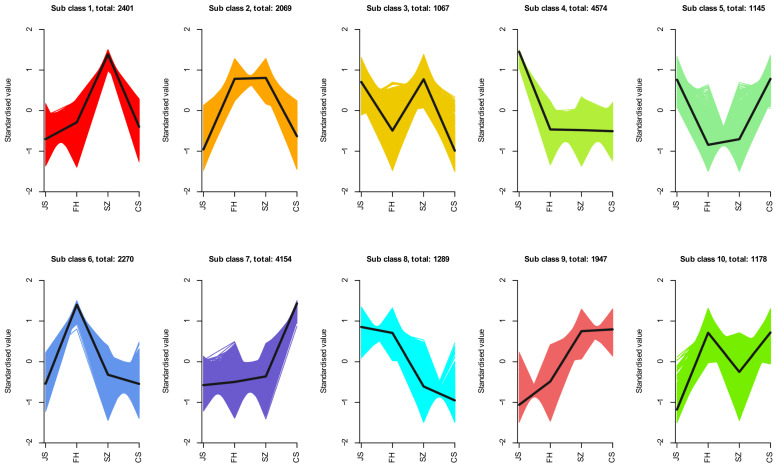
K-means clustering diagram of differential genes. Ten time-specific expression subclasses were resolved. Subclass 1 (2401 genes), peaking at SZ and enriched in growth-related pathways, represents a candidate subclass that may be involved in fruiting-body development; other subclasses display stage-specific or progressively increasing patterns.

K-means time-series clustering based on normalized gene expression levels divided all genes into ten subclasses (Figure 3). The horizontal axis represents the four developmental stage samples (JS, FH, SZ, and CS), and the vertical axis represents the normalized expression values. The solid black line indicates the average expression trend of all genes within each cluster, the colored shaded area represents the distribution range of expression levels across genes in the cluster, and the total number of genes in each subclass is indicated above the corresponding panel. The expression patterns were broadly consistent with the physiological characteristics of the four developmental stages of *P. nameko* (JS, FH, SZ, CS). Subclass 4 was specifically highly expressed at the JS stage and may be involved in the degradation and utilization of substrate nutrients. Subclasses 2 and 6 were upregulated at the FH stage and may be associated with basic developmental processes such as cell proliferation and cell wall synthesis. Subclass 1, containing 2401 genes, reached its expression peak only at the SZ stage, corresponding to the rapid growth stage of the fruiting body. Subclass 7 was continuously upregulated throughout development and may be involved in cap maturation and spore formation. GO and KEGG enrichment analyses revealed that Subclass 1 was significantly enriched in pathways related to rapid fruiting-body growth, while pathways associated with nutrient transport and cell expansion were distributed across multiple co-expressed subclasses, suggesting that Subclass 1, with its specific high expression at the SZ stage, represents a candidate subclass that may be involved in regulating fruiting-body growth and development in *P. nameko*.

### 3.3. Analysis of Differential Expressions

**Figure 4:**
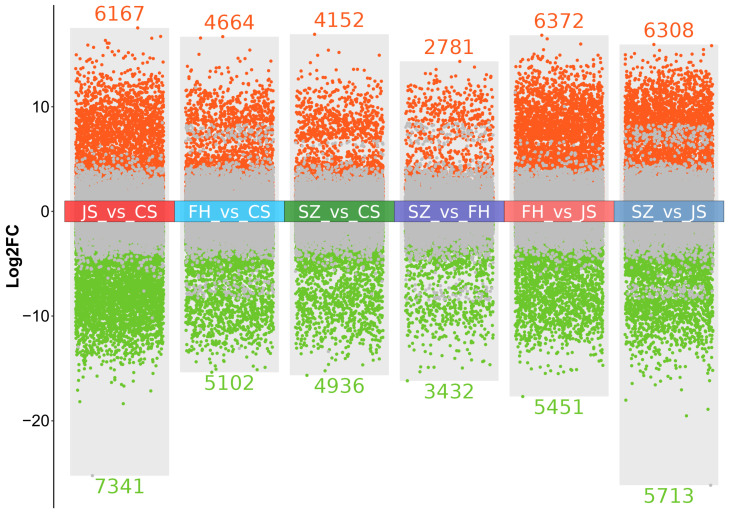
Volcano plots of DEGs from six pairwise comparisons. Red dots: upregulated genes; green dots: downregulated genes; gray dots: non-DEGs. Numbers mark the counts of up and downregulated genes in each group.

A volcano plot of differentially expressed genes (DEGs) from pairwise comparisons of four developmental stages of *P. nameko* (JS, FH, SZ, CS) is presented (Figure 4). DEGs were identified based on |log_2_ FC| ≥ 1 and FDR < 0.05; the vertical axis represents log_2_ FC, red dots indicate upregulated genes, green dots indicate downregulated genes, and gray dots represent non-DEGs. The numbers of DEGs in the six comparison groups are labeled above and below each plot, respectively: JS vs. CS, 13,508 DEGs (6167 upregulated, 7341 downregulated); FH vs. CS, 9766 DEGs (4664 upregulated, 5102 downregulated); SZ vs. CS, 9088 DEGs (4152 upregulated, 4936 downregulated); SZ vs. FH, 6213 DEGs (2781 upregulated, 3432 downregulated); FH vs. JS, 11,823 DEGs (6372 upregulated, 5451 downregulated); and SZ vs. JS, 12,021 DEGs (6308 upregulated, 5713 downregulated).

Substantial numbers of DEGs were detected in all comparison groups, with the highest number observed in the JS vs. CS group, indicating extensive transcriptome remodeling throughout the entire development of *P. nameko*. The number of DEGs across the six groups ranged from 6213 to 13,508, with the JS vs. CS comparison showing the highest (13,508), followed by SZ vs. JS (12,021) and FH vs. JS (11,823). This suggests that particularly extensive transcriptional reprogramming occurs during the transitions from the mycelial stage to the primordium stage and the subsequent growth phase, consistent with the biological requirement for coordinated expression of numerous genes during mycelium-to-primordium differentiation and rapid fruiting-body growth. Notably, in all comparison groups except FH vs. JS, the number of downregulated genes exceeded that of upregulated genes. In particular, the JS vs. CS comparison showed a roughly 16% higher number of downregulated genes (7341) than upregulated genes (6167), suggesting that gene suppression may play a dominant regulatory role during the transition from mycelium to fruiting-body development in *P. nameko*, a pattern especially prominent in the mycelium-versus-mature-stage comparisons. These marked transcriptional differences enabled further GO functional annotation, KEGG pathway enrichment analysis, and systematic identification of regulatory pathways and candidate genes involved in developmental transitions of *P. nameko*.

**Figure 5:**
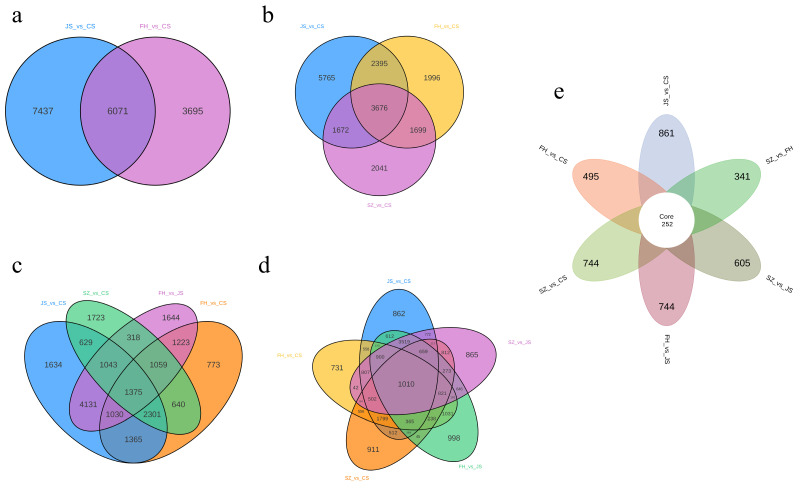
Venn diagram showing overlaps of differentially expressed genes (DEGs) from pairwise transcriptome comparisons. Different colors denote distinct comparison groups: blue = JS vs. CS, purple = FH vs. CS, yellow = SZ vs. CS, green = FH vs. JS, orange = SZ vs. JS, salmon = SZ vs. FH. (**a**) JS vs. CS and FH vs. CS; (**b**) JS vs. CS, FH vs. CS, and SZ vs. CS; (**c**) JS vs. CS, FH vs. CS, SZ vs. CS, and FH vs. JS; (**d**) JS vs. CS, FH vs. CS, SZ vs. CS, FH vs. JS, and SZ vs. JS; (**e**) Intersection of all six comparison groups.

A Venn diagram of differentially expressed genes (DEGs) from pairwise comparisons across the four developmental stages of *P. nameko* (JS, FH, SZ, CS) is presented (Figure 5), showing the number of DEG intersections among multiple comparison groups. As the number of included comparison groups increased, the total number of common DEGs gradually decreased: the intersection of JS vs. CS and FH vs. CS comprised 6071 DEGs; the intersection of JS vs. CS, FH vs. CS, and SZ vs. CS comprised 3676 DEGs; adding FH vs. JS yielded 1375 common DEGs; adding SZ vs. JS yielded 1010 common DEGs; and 252 DEGs were shared across all six comparison groups.

### 3.4. GO Functional Analysis of Genes with Differential Expression

The top 20 GO terms in each comparison group were selected for scatter plot visualization, with BP contributing 15 terms per group, CC contributing 2 terms per group with consistent second-level annotations, and MF contributing 15 terms per group, with differences mainly observed in molecular carrier activity, except for the SZ vs. FH comparison, where no MF variation was detected. For FH vs. CS (Figure S1), BP enrichment spanned six functional categories, including cellular and metabolic processes, with enrichments comprising oxidoreductase activity, peroxidase and antioxidant activity, oxidative stress response, unsaturated fatty acid metabolism and synthesis, nucleoside diphosphate synthesis, and protein dimerization. FH vs. JS (Figure S2) showed MF variation in multi-molecular carrier activity, with enrichments in oxidoreductase activity, processes involving oxygen-containing compounds, DNA-binding transcription factor activity, RNA polymerase II-related functions, and sequence-specific DNA binding. JS vs. CS (Figure S3) also exhibited MF variation in multi-molecular carrier activity, with enrichments in fungal cell wall polysaccharide metabolism, outer envelope structure, amide binding, redox enzyme activity, and nucleoprotein localization regulation. SZ vs. CS (Figure S4) displayed MF variation in multi-molecular carrier activity, with enrichments in unfolded protein binding, protein folding and assembly, and exopeptidase activity. SZ vs. FH (Figure S5) showed entirely consistent BP, CC and MF second-level terms, with enrichments including nucleoside diphosphate synthesis, acyl-CoA metabolism, manganese ion binding, proteasome complex, DNA replication initiation, and pigment synthesis and metabolism. SZ vs. JS (Figure S6) exhibited MF variation in multi-molecular carrier activity, with enrichments in oxidoreductase activity, bud neck, response to oxidized substances, transcription factor activity, vesicular transport, and Golgi-associated structures.

The GO terms across all comparison groups collectively suggest developmental processes of *P. nameko*: redox and biosynthetic pathways provide energy for growth at each stage and maintain cellular homeostasis; DNA replication, cell wall metabolism, and substance transport regulate cell proliferation and fruiting-body morphogenesis; and differences in multiple molecular carrier activities reflect dynamic changes in molecular interactions during development. Notably, the functional patterns of the SZ and FH stages are similar, and the overall enrichment results suggest the molecular regulatory basis underlying the entire growth and development cycle of *P. nameko*.

### 3.5. KEGG Analysis of Genes with Differential Expression

**Figure 6:**
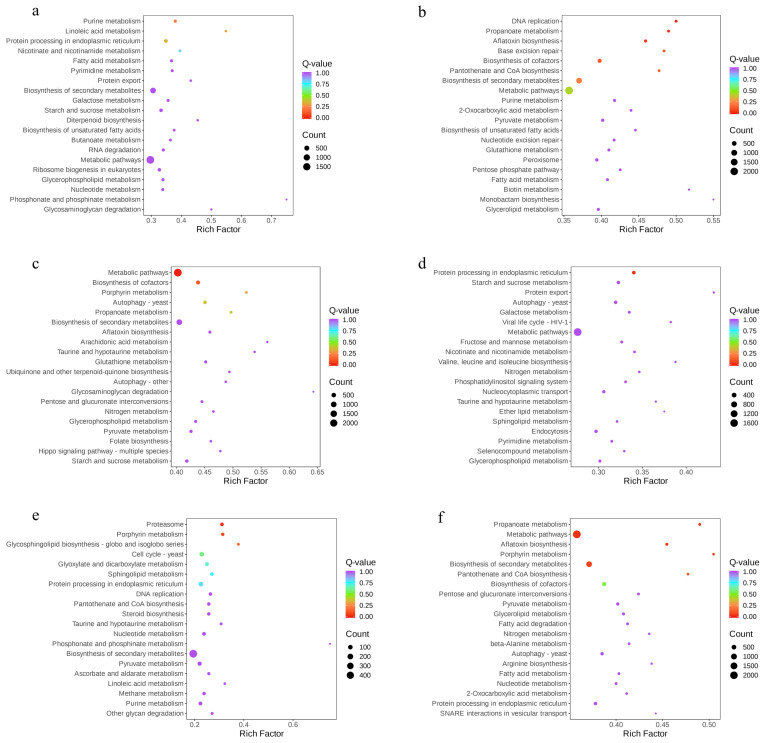
KEGG enrichment scatter plot of differentially expressed genes in each group. The figure is split into six subfigures (**a**–**f**) for FH vs. CS, FH vs. JS, JS vs. CS, SZ vs. CS, SZ vs. FH, and SZ vs. JS, each showing the top 20 KEGG pathways. Enriched pathways include purine and linoleic acid metabolism, DNA repair, proteasome, porphyrin metabolism, ER protein processing, starch and sucrose metabolism, and cofactor and CoA biosynthesis, reflecting stage-specific metabolic reprogramming during development.

The KEGG database was used to annotate differentially expressed genes, and scatter plots were produced for the top 20 pathways. The enrichment patterns for each comparison group were as follows: FH vs. CS (Figure 6a): significant enrichment in purine metabolism and linoleic acid metabolism, with broad enrichment in secondary metabolite synthesis and galactose metabolism; FH vs. JS (Figure 6b): highly enriched in coenzyme synthesis and secondary metabolite synthesis, with significant enrichment in DNA repair and propionate metabolism; JS vs. CS (Figure 6c): significantly and highly enriched in porphyrin metabolism, coenzyme synthesis, and metabolic pathways; SZ vs. CS (Figure 6d): enriched in protein export, starch and sucrose metabolism, and endoplasmic reticulum protein processing; SZ vs. FH (Figure 6e): enriched in proteasome, porphyrin metabolism, and sphingolipid synthesis; SZ vs. JS (Figure 6f): enriched in porphyrin metabolism, secondary metabolite synthesis, and pantothenic acid and coenzyme A synthesis KEGG pathways across all comparison groups may be associated with metabolic, cellular proliferation, and protein processing processes at different developmental stages of *P. nameko*: purine, carbohydrate, lipid, and coenzyme synthesis pathways may contribute to mycelial growth, primordium differentiation, and fruiting-body expansion; DNA repair and proteasome pathways may be involved in cell division and homeostasis; endoplasmic reticulum protein processing and protein export may contribute to fruiting-body cell construction, while porphyrin and secondary metabolism pathways may respond to developmental stage transitions, collectively suggesting the material metabolic regulatory network underlying the entire growth and development cycle of *P. nameko*.

### 3.6. Identification of Candidate Genes with Stage-Specific Expression Patterns

Based on transcriptome-wide differential expression analysis—JS, FH, SZ, and CS—only the FH and SZ stages showed similar gene expression patterns, while the other two stages exhibited notable differences, which is consistent with the growth and development pattern of *P. nameko*. This study examined the metabolic pathways where differentially expressed genes were significantly enriched in the FH vs. CS and SZ vs. CS groups, using the CS stage as a control. After the intersection of differentially expressed genes from the FH vs. CS and SZ vs. CS groups was found, genes were screened based on upregulation, log2FC ≥ 1, and FDR < 0.05, and a total of 13 genes that might be related to the growth and development of *P. nameko* were obtained, namely *Cluster-6756.10*, *Cluster-6756.6*, *Cluster-8242.2*, *Cluster-7158.6*, *Cluster-6021.12*, *Cluster-6641.3*, *Cluster-2676.1*, *Cluster-7065.16*, *Cluster-7415.13*, *Cluster-5044.5*, *Cluster-8234.0*, *Cluster-8028.4* and *Cluster-7437.5*.

### 3.7. RT-qPCR Validation of Transcriptome Sequencing Data

#### 3.7.1. Sequences of RT-qPCR Primers

The primer sequences for the internal reference gene and the 13 genes associated with growth and development in *P. nameko* are listed in Table 1. To identify a stable internal reference gene for RT-qPCR across the four developmental stages of *P. nameko*, four candidate housekeeping genes were evaluated using a combination of geNorm, NormFinder, and BestKeeper. *EF1α-1* satisfied all the criteria, including M < 1.5, minimum SV, SD < 1, and no significant variation in Ct values across the four stages (*p* > 0.05). Furthermore, the pairwise variation value V2 and V3 calculated by geNorm was below 0.15, indicating that *EF1α-1* alone is sufficient for normalization of expression levels.

#### 3.7.2. 13 Genes’ Expression Analysis

**Figure 7:**
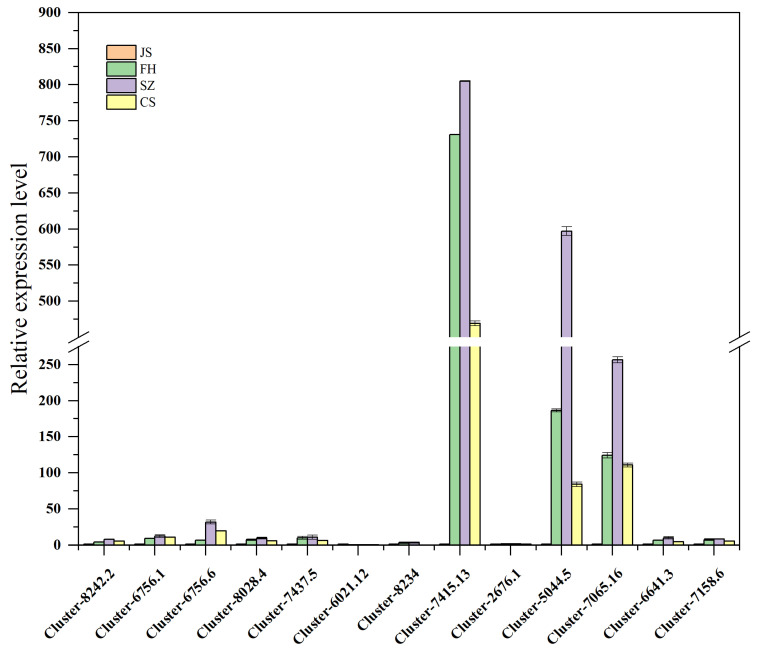
Relative expression levels of the gene. RT-qPCR analysis of 13 candidate genes across four developmental stages (JS, FH, SZ, CS).

The 13 genes that might be associated with the growth and development of *P. nameko* were subjected to RT-qPCR analysis. As shown in the results, the gene expression levels across the four developmental stages of *P. nameko* followed a trend of SZ > FH > CS > JS, which is consistent with the actual growth pattern (Figure 7). Among these genes, *Cluster-7415.13* exhibited significantly higher expression levels than the other candidate genes throughout the four developmental stages, and its expression timing showed a pattern coincident with fruiting-body morphogenesis. Furthermore, it is speculated that this gene may be involved throughout mycelial nutrient accumulation, primordium differentiation, fruiting-body expansion, and maturation development, and may represent a candidate gene with potential relevance to the growth and development of *P. nameko*.

## 4. Discussion

The vegetative growth stage of *P. nameko*’s entire life cycle is the mycelial stage. During this phase, the mycelium is in a state of vigorous growth, with intense protein biosynthesis, synthesis of various carbohydrates, and replication of genetic material [27,44]. After the cultivation temperature is adjusted, the mycelium transitions from vegetative to reproductive growth. As the mycelium aggregates to form primordia and differentiates into the initial fruiting-body morphology, cellular processes such as purine metabolism [24], linoleic acid metabolism [45], and protein processing in the endoplasmic reticulum [46] become active. Differentially expressed genes are significantly enriched in the biosynthesis of secondary metabolites [47], galactose metabolism, and starch and sucrose metabolism [48,49,50]. As the fruiting body develops and matures, just before the mature stage, the growth rate reaches its peak. At this point, intracellular carbohydrate synthesis, lipid metabolism, cofactor and vitamin metabolism, energy metabolism, transport and catabolism, and amino acid metabolism appear to be transcriptionally active [51]. When the pileus opens and spores are expelled, the fruiting body reaches maturity. At this stage, genes that showed high expression levels during the growth phase are downregulated, growth stops, and the life cycle of *P. nameko* is completed [52].

In our practical observations, we found that *P. nameko* secretes a mucus-like substance on the cap surface, which is viscous, adhesive, and cool to the touch. Slime secretion follows a cyclical pattern: absent during the mycelial stage, initiated at the primordium differentiation stage, reaching a peak during the growth stage, and then gradually declining until cessation at maturity. This pattern is consistent with the developmental cycle of *P. nameko*. Therefore, the process of slime secretion can be considered a stage-specific trait that appears to coincide with reproductive development. The secretion dynamics are synchronized with fruiting-body growth and may be associated with developmental processes, such as rapid fruiting-body expansion and primordium differentiation. As such, slime appears to be a characteristic physiological product associated with the completion of the entire life cycle of *P. nameko*. Currently, this study has preliminarily screened genes that may be closely associated with the growth and development of *P. nameko*; however, due to the species-specific characteristics of *P. nameko*, functional validation of these genes remains in the exploratory stage.

## 5. Conclusions

In this study, transcriptome sequencing was performed on four developmental stages of *P. nameko* (JS, FH, SZ, and CS) to screen for differentially expressed genes, followed by GO and KEGG functional enrichment analyses. The results showed that DEGs in the FH vs. CS and SZ vs. CS comparison groups were primarily enriched in pathways related to protein processing, fatty acid metabolism, and linoleic acid metabolism, suggesting that alterations in these specific metabolic pathways may be closely associated with the growth and development of *P. nameko*. Using the CS stage as a reference, the intersection of DEGs from the FH vs. CS and SZ vs. CS groups was obtained, and genes were screened based on upregulation, log_2_ FC ≥ 1, and FDR < 0.05, yielding a total of 13 candidate genes. RT-qPCR analysis revealed that the expression levels of these genes across the four developmental stages followed a descending order of SZ > FH > CS > JS, which is consistent with the actual growth pattern. Among them, *Cluster-7415.13* exhibited the highest expression levels across all four stages, suggesting that this gene may be potentially involved in regulating the growth and development of *P. nameko*. This study provides insights into the transcriptomic dynamics underlying the development of *P. nameko* and offers a valuable resource of candidate genes for future functional validation.

## Figures and Tables

**Table 1:** Primer sequences of RT-qPCR.

Gene	Forward Primer Sequence	Reverse Primer Sequence
*Cluster-8242.2*	CCCCGTTCACTTTCTCTGCT	ACGGAACAGAGGCACAGAAG
*Cluster-6756.1*	TTCTCGTTCGTGTCCCACTG	TCGGAAACAGGCTGATGGTC
*Cluster-6756.6*	ATCTCCTTCCTCCCCACCTC	CGCTTCGACTCCGCTAGATT
*Cluster-7158.6*	CGAACCTGGTATCTACCCGC	GAAGCTGACACGACTCTGCT
* Cluster-6021.12 *	GCGCGAAGGAAAATCGTCTC	TTCGTTCGTCGTAGTGTGCA
*Cluster-6641.3*	AGCCAAAAGTTCGTCAACGC	AGTGCTACTTCCGACGCAAA
*Cluster-2676.1*	TCCTTGTCGGGGGTGATACT	CCAACTGCTCCTATGCCCTC
*Cluster-7065.16*	TTTCGAAGTCCATGCCTCCC	ATGGGCGGTATGGGAGGTAT
*Cluster-7415.13*	TCGTTCTTGGAGAGGGCAAC	TCGTCCCCAAACTTGAGACG
*Cluster-5044.5*	CCTTCGGACTCGGATGTACG	TGCCCCTATTCTCATCCCCA
*Cluster-8234.0*	CGACGACTGTAGGATTCCCG	TTCAGCTCGTTGAGGACACC
*Cluster-7437.5*	GACTGAGTACAGCGGCTTGT	CTGCACGATTTCCAGCGATG
*Cluster-8028.4*	AGCTCGGAGTTAAGCAGCAG	TACACAGCGTCATAGGTGCC
*EF1a-1*	CAACTTCGCCCCTTCAAACG	GTTGAAGCCGACGTTGTCAC

## Data Availability

Raw transcriptome sequencing data generated in this study are openly available in NCBI Sequence Read Archive (SRA) under Bio Project accession number PRJNA1497984.

## Supplementary Materials

The following supporting information can be downloaded at https://www.mdpi.com/article/10.3390/jof12070542/s1, Supplementary Material File S1 (See in Supplementary PDF). Figure S1. GO enrichment scatter plot and secondary classification diagram of differentially expressed genes in the FH vs. CS comparison group. The top 20 significantly enriched GO terms are presented. Figure S2. GO enrichment scatter plot and secondary classification diagram of differentially expressed genes in the FH vs. JS comparison group. The top 20 significantly enriched GO terms are presented. Figure S3. GO enrichment scatter plot and secondary classification diagram of differentially expressed genes in the JS vs. CS comparison group. The top 20 significantly enriched GO terms are presented. Figure S4. GO enrichment scatter plot and secondary classification diagram of differentially expressed genes in the SZ vs. CS comparison group. The top 20 significantly enriched GO terms are presented. Figure S5. GO enrichment scatter plot and secondary classification diagram of differentially expressed genes in the SZ vs. FH comparison group. The top 20 significantly enriched GO terms are presented. Figure S6. GO enrichment scatter plot and secondary classification diagram of differentially expressed genes in the SZ vs. JS comparison group. The top 20 significantly enriched GO terms are presented.

## Abbreviations

The following abbreviations are used in this manuscript:

*Pholiota nameko*	*P. nameko*
GO	Gene Ontology
KEGG	Kyoto Encyclopedia of Genes and Genomes
RT-qPCR	Quantitative real-time PCR
JS	The mycelial stage
FH	The primordium stage
SZ	The growth stage
CS	The maturity stage
