# Supplementary material for: Transcriptomic Profiling of Developmental Stages and Screening of Candidate Genes in Pholiota nameko
Source: J Fungi (Basel). 2026 Jul 22;12(7):542. doi: 10.3390/jof12070542 (PMC13412918; doi:10.3390/jof12070542)
Supplement: Supplementary file 1 [file jof-12-00542-s001.zip › jof-4359808-supplementary.pdf]

Supplementary Material File S1

Figure Legends

Figure S1. GO enrichment scatter plot and secondary classification diagram of DEGs in FH vs CS. The top 20 significantly enriched GO terms are presented.

Figure S2. GO enrichment scatter plot and secondary classification diagram of DEGs in FH vs JS. The top 20 significantly enriched GO terms are presented.

Figure S3. GO enrichment scatter plot and secondary classification diagram of DEGs in JS vs CS. The top 20 significantly enriched GO terms are presented.

Figure S4. GO enrichment scatter plot and secondary classification diagram of DEGs in SZ vs CS. The top 20 significantly enriched GO terms are presented.

Figure S5. GO enrichment scatter plot and secondary classification diagram of DEGs in SZ vs FH. The top 20 significantly enriched GO terms are presented.

Figure S6. GO enrichment scatter plot and secondary classification diagram of DEGs in SZ vs JS. The top 20 significantly enriched GO terms are presented.

Figure S1.

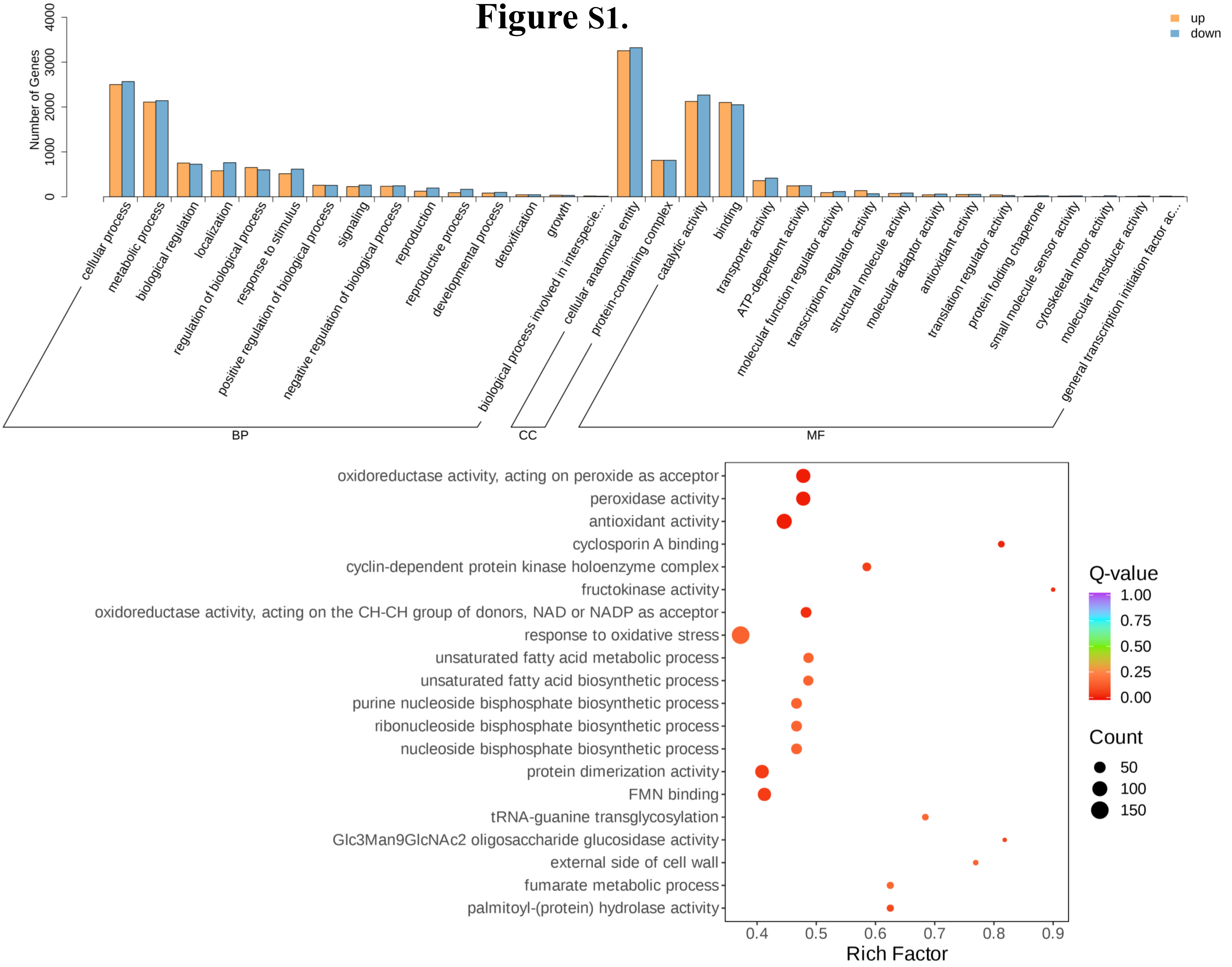

up
down
Number of Genes
0
1000
2000
3000
4000
cellular process
metabolic process
biological regulation
localization
regulation of biological process
response to stimulus
positive regulation of biological process
signaling
negative regulation of biological process
reproduction
reproductive process
developmental process
detoxification
growth
biological process involved in interspecie...
cellular anatomical entity
protein-containing complex
catalytic activity
binding
transporter activity
ATP-dependent activity
molecular function regulator activity
transcription regulator activity
structural molecule activity
molecular adaptor activity
antioxidant activity
translation regulator activity
protein folding chaperone
small molecule sensor activity
cytoskeletal motor activity
molecular transducer activity
general transcription initiation factor ac...
BP
CC
MF
oxidoreductase activity, acting on peroxide as acceptor
peroxidase activity
antioxidant activity
cyclosporin A binding
cyclin-dependent protein kinase holoenzyme complex
fructokinase activity
oxidoreductase activity, acting on the CH-CH group of donors, NAD or NADP as acceptor
response to oxidative stress
unsaturated fatty acid metabolic process
unsaturated fatty acid biosynthetic process
purine nucleoside bisphosphate biosynthetic process
ribonucleoside bisphosphate biosynthetic process
nucleoside bisphosphate biosynthetic process
protein dimerization activity
FMN binding
tRNA-guanine transglycosylation
Glc3Man9GlcNAc2 oligosaccharide glucosidase activity
external side of cell wall
fumarate metabolic process
palmitoyl-(protein) hydrolase activity
0.4
0.5
0.6
0.7
0.8
0.9
Rich Factor
Q-value
1.00
0.75
0.50
0.25
0.00
Count
50
100
150

Figure S2.

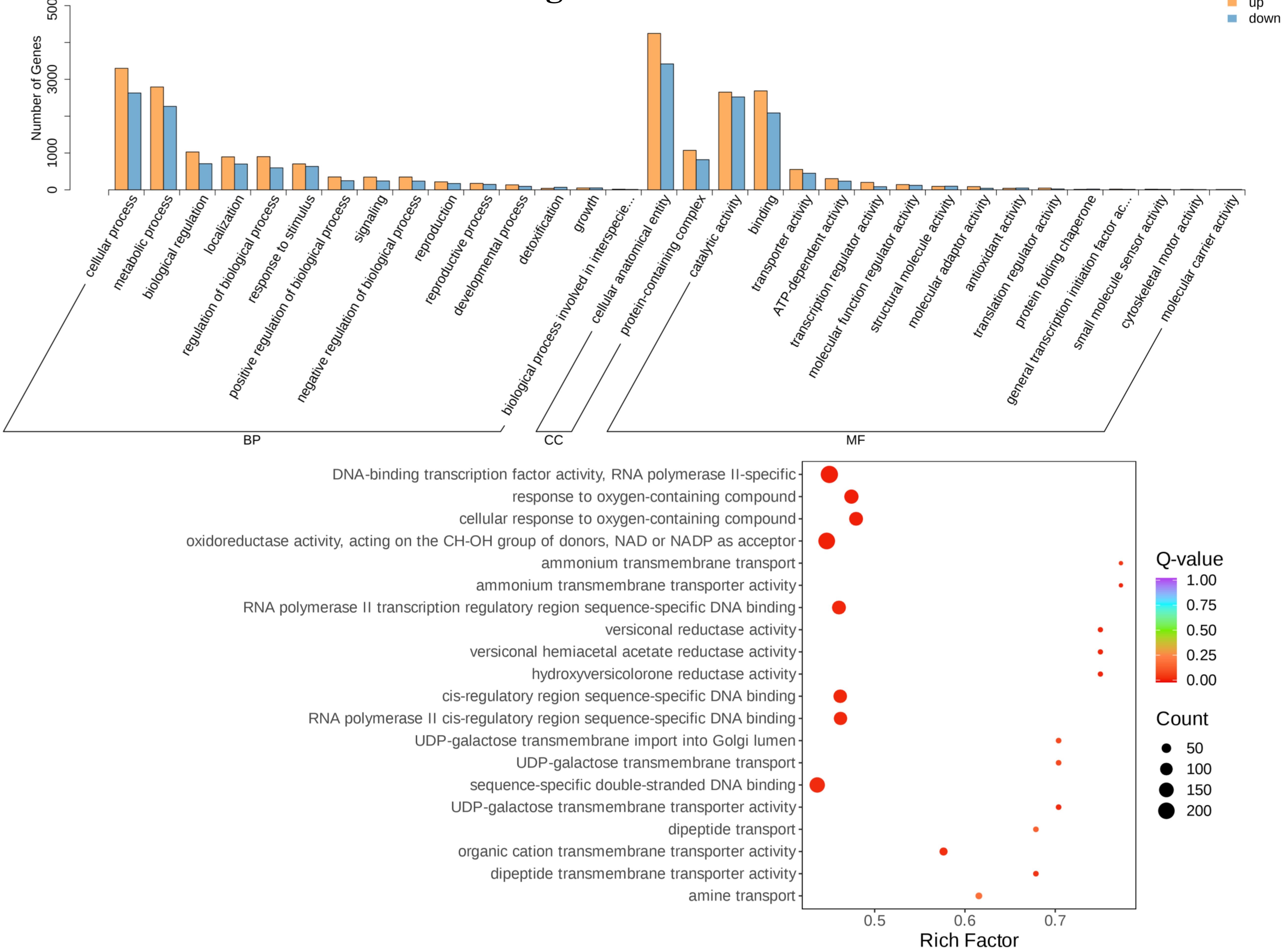
up
down
Number of Genes
0
1000
3000
5000
cellular process
metabolic process
biological regulation
localization
regulation of biological process
response to stimulus
positive regulation of biological process
signaling
negative regulation of biological process
reproduction
reproductive process
developmental process
detoxification
growth
biological process involved in interspecie...
cellular anatomical entity
protein-containing complex
catalytic activity
binding
transporter activity
ATP-dependent activity
transcription regulator activity
molecular function regulator activity
structural molecule activity
molecular adaptor activity
antioxidant activity
translation regulator activity
protein folding chaperone
general transcription initiation factor ac...
small molecule sensor activity
cytoskeletal motor activity
molecular carrier activity
BP
CC
MF
DNA-binding transcription factor activity, RNA polymerase II-specific
response to oxygen-containing compound
cellular response to oxygen-containing compound
oxidoreductase activity, acting on the CH-OH group of donors, NAD or NADP as acceptor
ammonium transmembrane transport
ammonium transmembrane transporter activity
RNA polymerase II transcription regulatory region sequence-specific DNA binding
versiconal reductase activity
versiconal hemiacetal acetate reductase activity
hydroxyversicolorone reductase activity
cis-regulatory region sequence-specific DNA binding
RNA polymerase II cis-regulatory region sequence-specific DNA binding
UDP-galactose transmembrane import into Golgi lumen
UDP-galactose transmembrane transport
sequence-specific double-stranded DNA binding
UDP-galactose transmembrane transporter activity
dipeptide transport
organic cation transmembrane transporter activity
dipeptide transmembrane transporter activity
amine transport
0.5
0.6
0.7
Rich Factor
Q-value
1.00
0.75
0.50
0.25
0.00
Count
50
100
150
200

Figure S3.

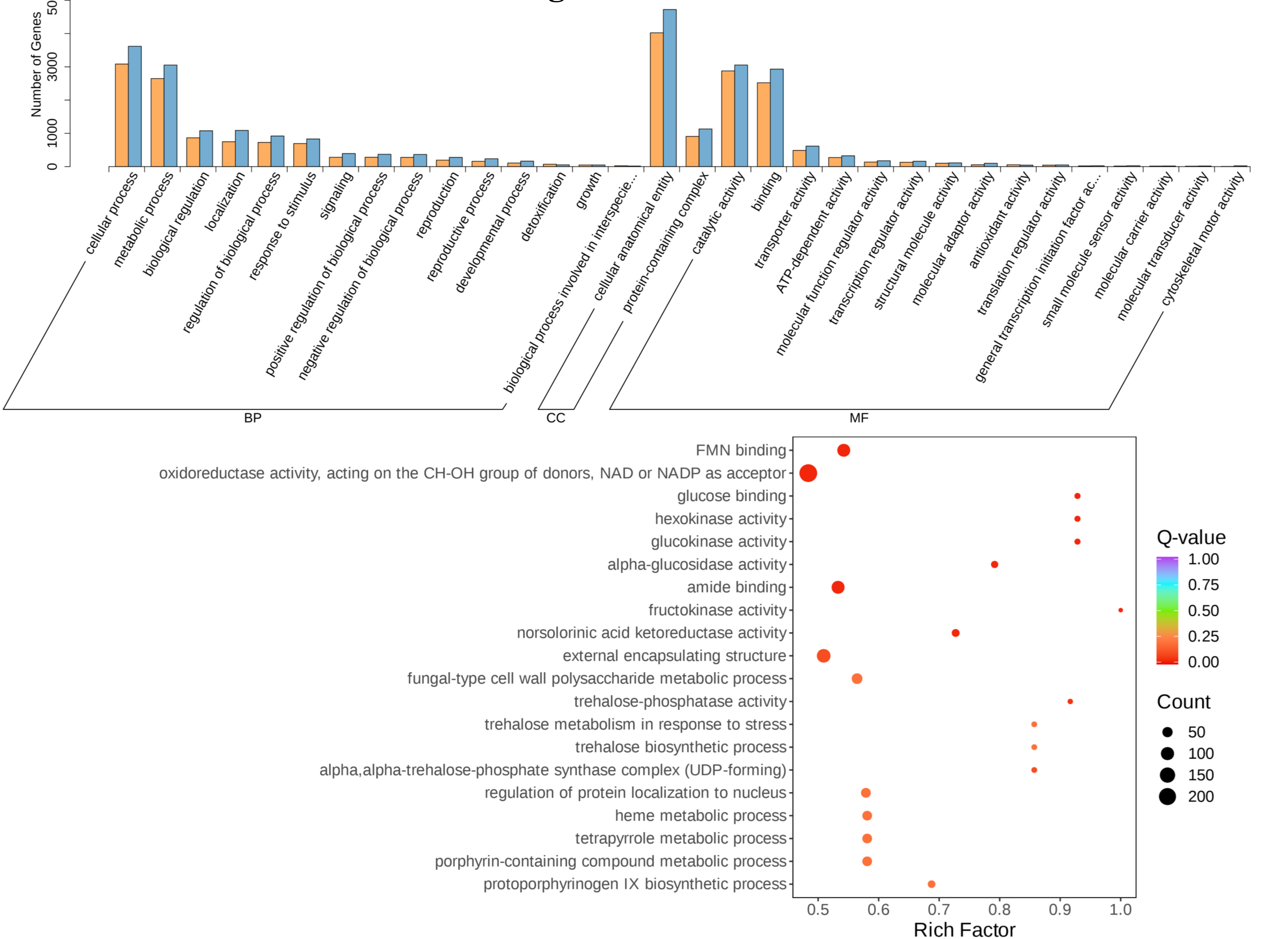

up
down
Number of Genes
0
1000
3000
5000
cellular process
metabolic process
biological regulation
localization
regulation of biological process
response to stimulus
signaling
positive regulation of biological process
negative regulation of biological process
reproduction
reproductive process
developmental process
detoxification
growth
biological process involved in interspecie...
cellular anatomical entity
protein-containing complex
catalytic activity
binding
transporter activity
ATP-dependent activity
molecular function regulator activity
transcription regulator activity
structural molecule activity
molecular adaptor activity
antioxidant activity
translation regulator activity
general transcription initiation factor ac...
small molecule sensor activity
molecular carrier activity
molecular transducer activity
cytoskeletal motor activity
BP
CC
MF
FMN binding
oxidoreductase activity, acting on the CH-OH group of donors, NAD or NADP as acceptor
glucose binding
hexokinase activity
glucokinase activity
alpha-glucosidase activity
amide binding
fructokinase activity
norsolorinic acid ketoreductase activity
external encapsulating structure
fungal-type cell wall polysaccharide metabolic process
trehalose-phosphatase activity
trehalose metabolism in response to stress
trehalose biosynthetic process
alpha,alpha-trehalose-phosphate synthase complex (UDP-forming)
regulation of protein localization to nucleus
heme metabolic process
tetrapyrrole metabolic process
porphyrin-containing compound metabolic process
protoporphyrinogen IX biosynthetic process
0.5
0.6
0.7
0.8
0.9
1.0
Rich Factor
Q-value
1.00
0.75
0.50
0.25
0.00
Count
50
100
150
200

Figure S4.

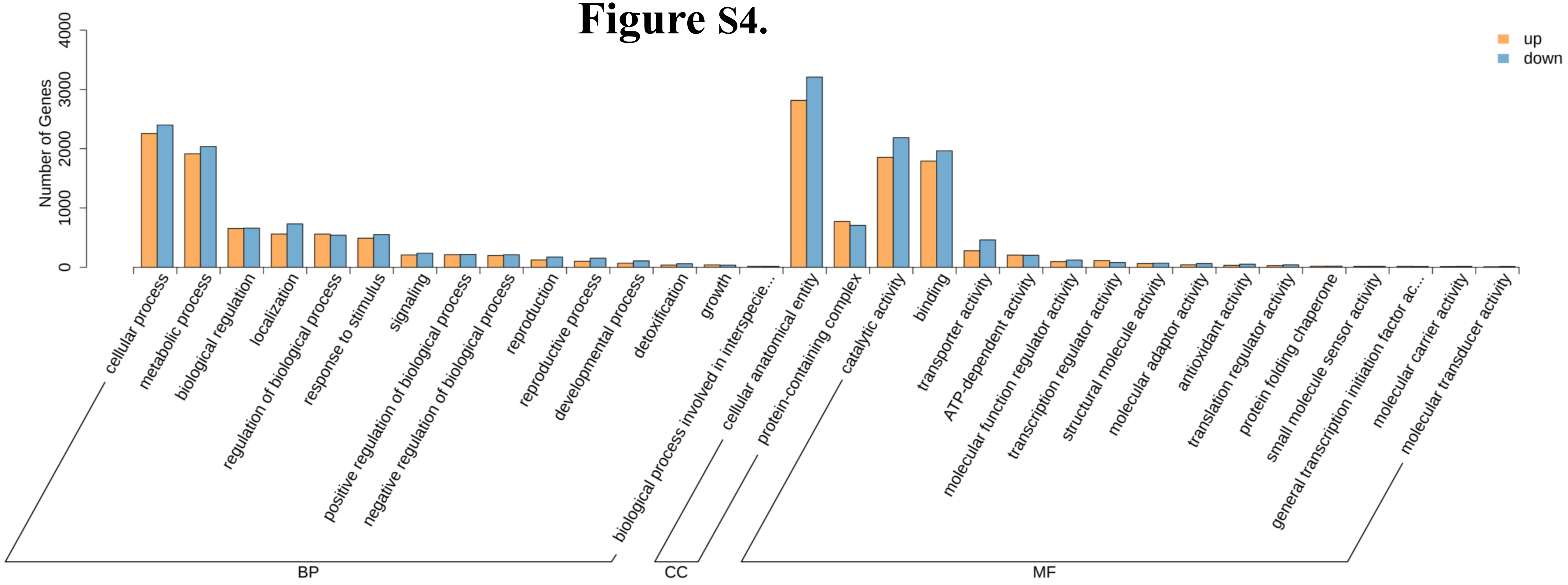
up
down
Number of Genes
0
1000
2000
3000
4000
cellular process
metabolic process
biological regulation
localization
regulation of biological process
response to stimulus
signaling
positive regulation of biological process
negative regulation of biological process
reproduction
reproductive process
developmental process
detoxification
growth
biological process involved in interspecie...
cellular anatomical entity
protein-containing complex
catalytic activity
binding
transporter activity
ATP-dependent activity
molecular function regulator activity
transcription regulator activity
structural molecule activity
molecular adaptor activity
antioxidant activity
translation regulator activity
protein folding chaperone
small molecule sensor activity
general transcription initiation factor ac...
molecular carrier activity
molecular transducer activity
BP
CC
MF

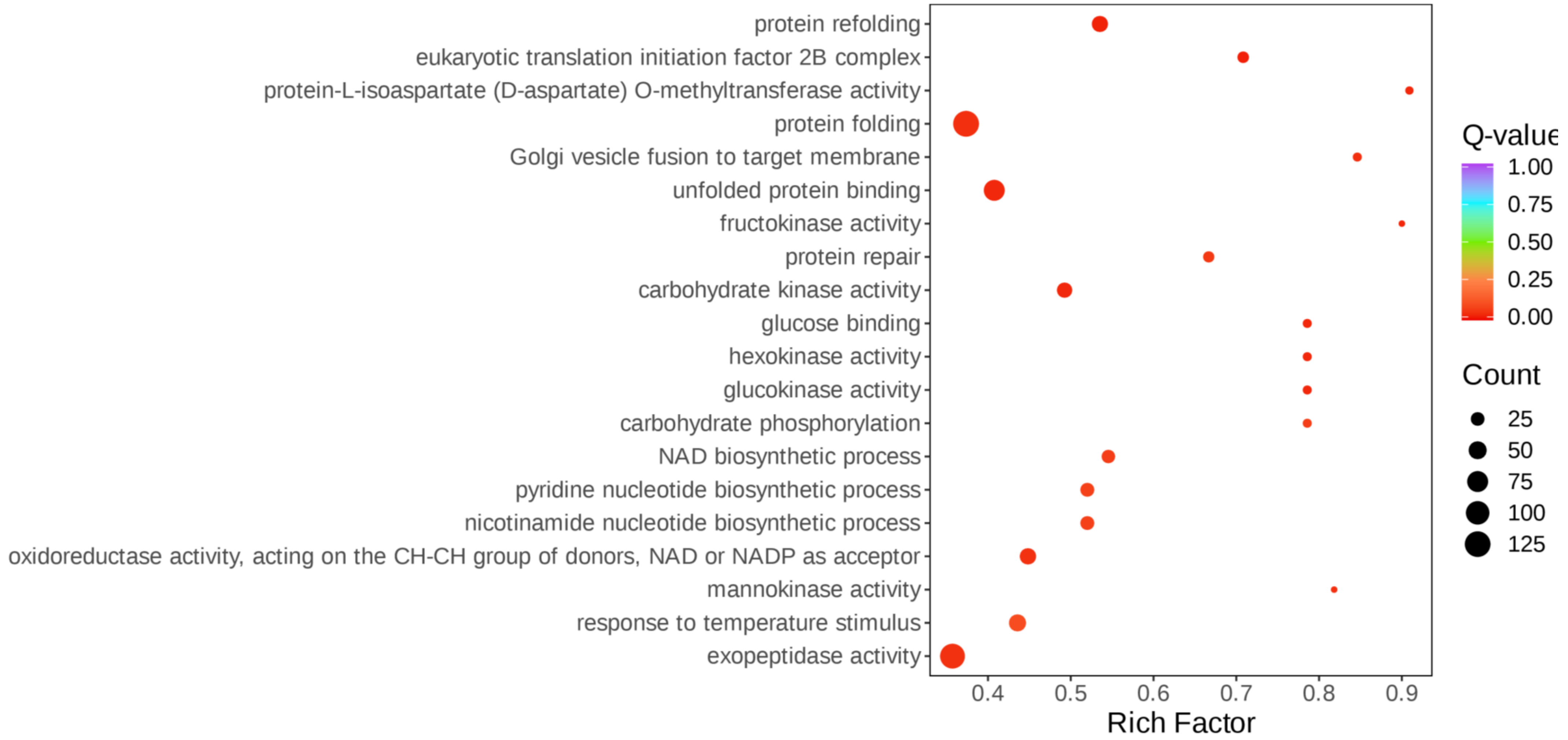
protein refolding
eukaryotic translation initiation factor 2B complex
protein-L-isoaspartate (D-aspartate) O-methyltransferase activity
protein folding
Golgi vesicle fusion to target membrane
unfolded protein binding
fructokinase activity
protein repair
carbohydrate kinase activity
glucose binding
hexokinase activity
glucokinase activity
carbohydrate phosphorylation
NAD biosynthetic process
pyridine nucleotide biosynthetic process
nicotinamide nucleotide biosynthetic process
oxidoreductase activity, acting on the CH-CH group of donors, NAD or NADP as acceptor
mannokinase activity
response to temperature stimulus
exopeptidase activity
0.4
0.5
0.6
0.7
0.8
0.9
Rich Factor
Q-value
1.00
0.75
0.50
0.25
0.00
Count
25
50
75
100
125

Figure S5.

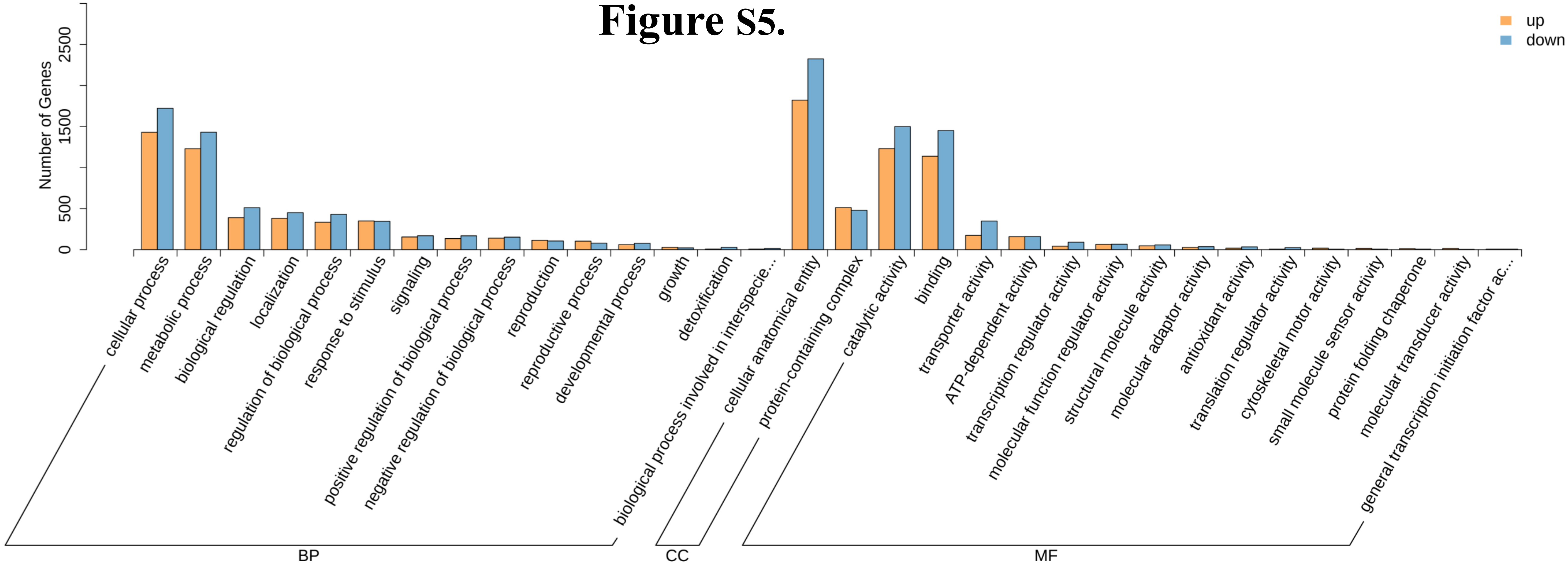

up
down
Number of Genes
0
500
1500
2500
cellular process
metabolic process
biological regulation
localization
regulation of biological process
response to stimulus
signaling
positive regulation of biological process
negative regulation of biological process
reproduction
reproductive process
developmental process
growth
detoxification
biological process involved in interspecie...
cellular anatomical entity
protein-containing complex
catalytic activity
binding
transporter activity
ATP-dependent activity
transcription regulator activity
molecular function regulator activity
structural molecule activity
molecular adaptor activity
antioxidant activity
translation regulator activity
cytoskeletal motor activity
small molecule sensor activity
protein folding chaperone
molecular transducer activity
general transcription initiation factor ac...
BP
CC
MF

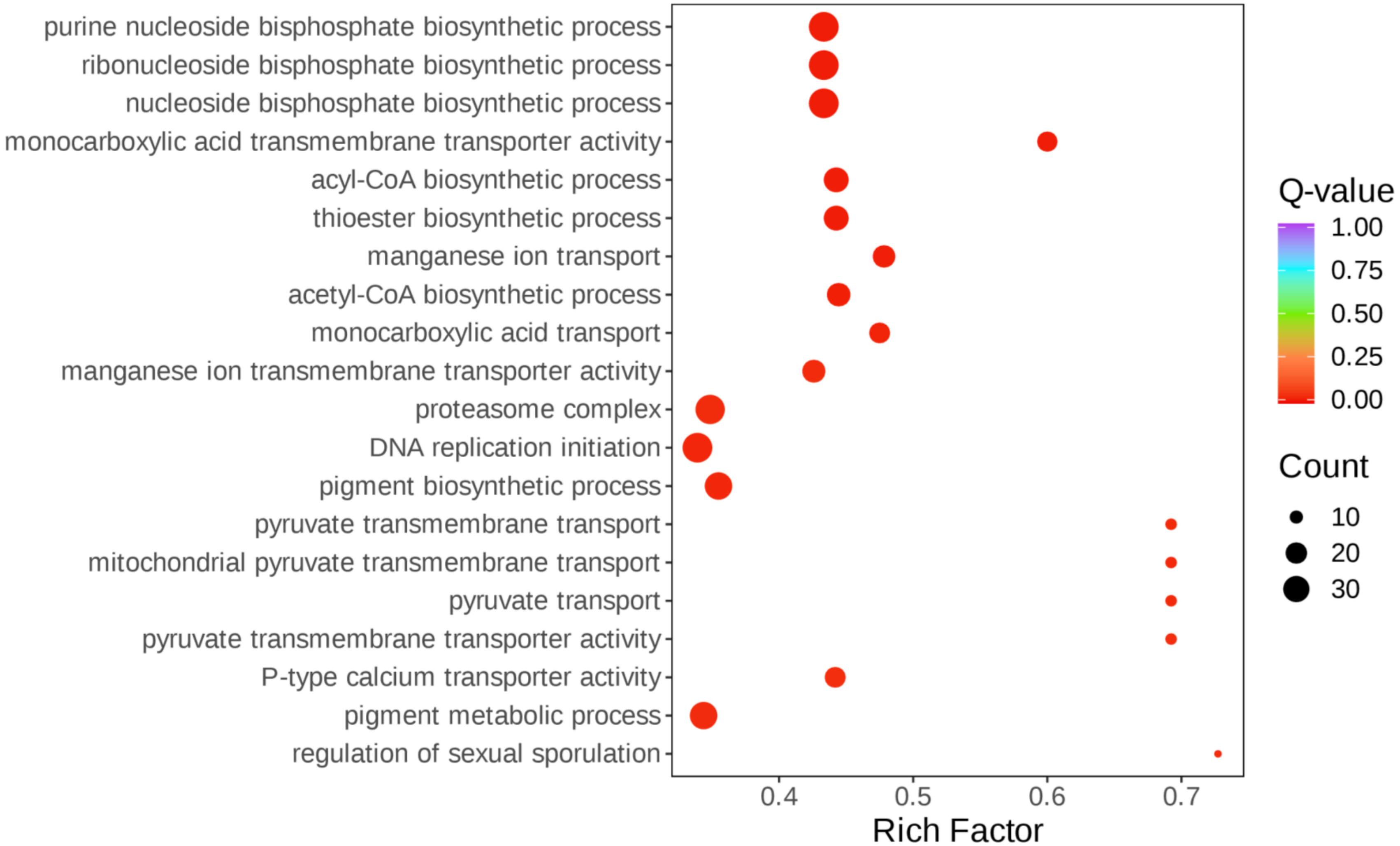

purine nucleoside bisphosphate biosynthetic process
ribonucleoside bisphosphate biosynthetic process
nucleoside bisphosphate biosynthetic process
monocarboxylic acid transmembrane transporter activity
acyl-CoA biosynthetic process
thioester biosynthetic process
manganese ion transport
acetyl-CoA biosynthetic process
monocarboxylic acid transport
manganese ion transmembrane transporter activity
proteasome complex
DNA replication initiation
pigment biosynthetic process
pyruvate transmembrane transport
mitochondrial pyruvate transmembrane transport
pyruvate transport
pyruvate transmembrane transporter activity
P-type calcium transporter activity
pigment metabolic process
regulation of sexual sporulation
0.4
0.5
0.6
0.7
Rich Factor
Q-value
1.00
0.75
0.50
0.25
0.00
Count
10
20
30

Figure S6.

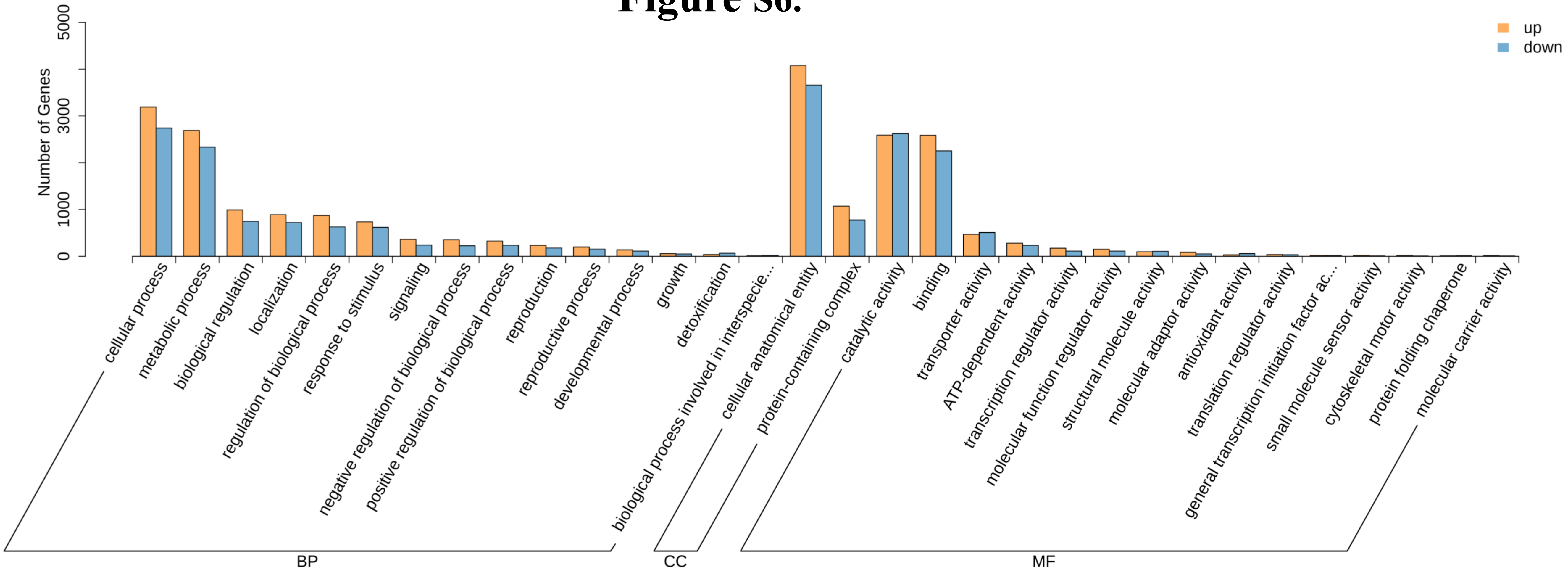

up
down
Number of Genes
0
1000
3000
5000
cellular process
metabolic process
biological regulation
localization
regulation of biological process
response to stimulus
signaling
negative regulation of biological process
positive regulation of biological process
reproduction
reproductive process
developmental process
growth
detoxification
biological process involved in interspecie...
cellular anatomical entity
protein-containing complex
catalytic activity
binding
transporter activity
ATP-dependent activity
transcription regulator activity
molecular function regulator activity
structural molecule activity
molecular adaptor activity
antioxidant activity
translation regulator activity
general transcription initiation factor ac...
small molecule sensor activity
cytoskeletal motor activity
protein folding chaperone
molecular carrier activity
BP
CC
MF

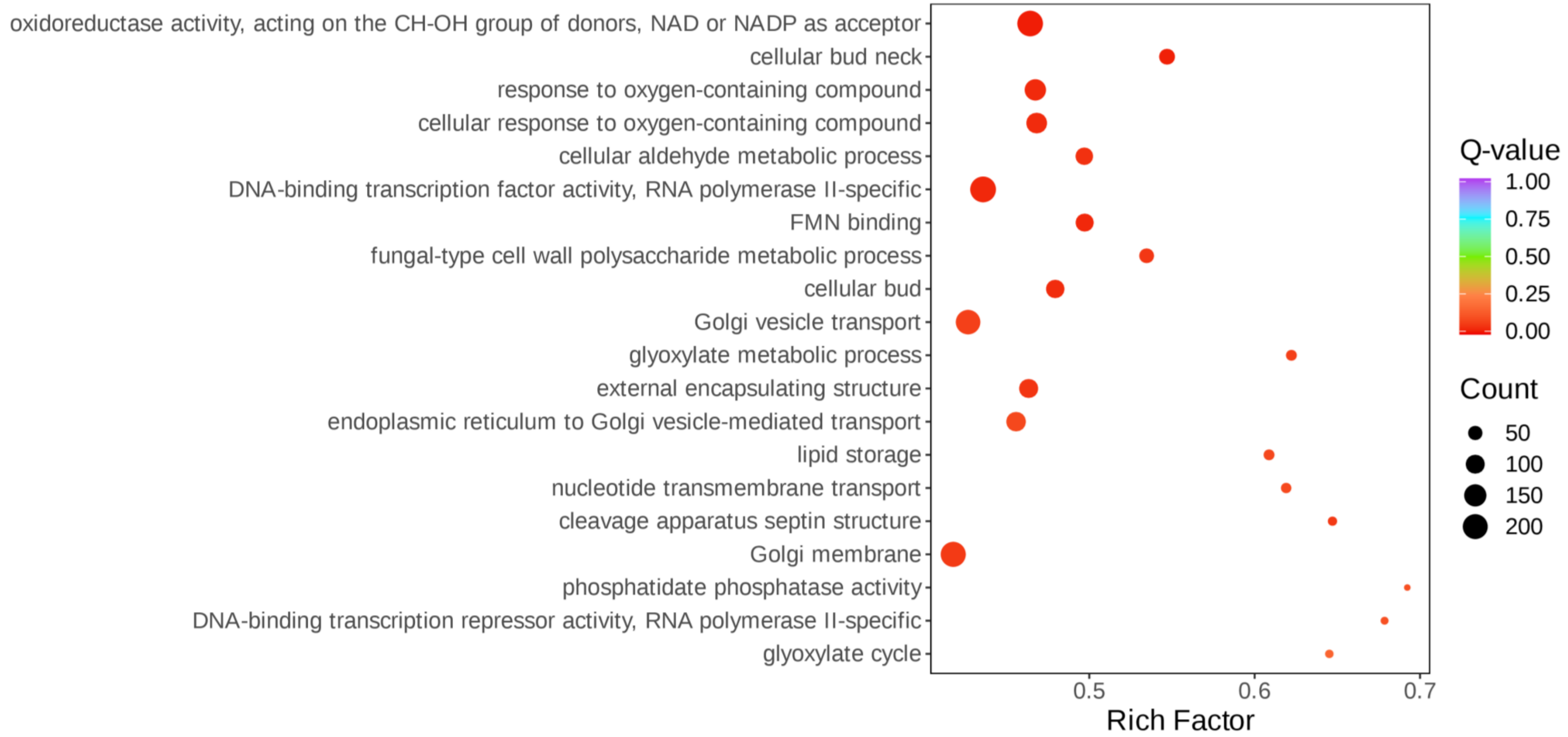

oxidoreductase activity, acting on the CH-OH group of donors, NAD or NADP as acceptor
cellular bud neck
response to oxygen-containing compound
cellular response to oxygen-containing compound
cellular aldehyde metabolic process
DNA-binding transcription factor activity, RNA polymerase II-specific
FMN binding
fungal-type cell wall polysaccharide metabolic process
cellular bud
Golgi vesicle transport
glyoxylate metabolic process
external encapsulating structure
endoplasmic reticulum to Golgi vesicle-mediated transport
lipid storage
nucleotide transmembrane transport
cleavage apparatus septin structure
Golgi membrane
phosphatidate phosphatase activity
DNA-binding transcription repressor activity, RNA polymerase II-specific
glyoxylate cycle
0.5
0.6
0.7
Rich Factor
Q-value
1.00
0.75
0.50
0.25
0.00
Count
50
100
150
200
